# Single-Blind, Prospective, Randomized Study of Cefmetazole and Cefoxitin in the Treatment of Postcesarean Endometritis

**DOI:** 10.1155/S1064744995000263

**Published:** 1995

**Authors:** Ashwin Chatwani, Mark Martens, David A. Grimes, Molly Chatterjee, Melvin Noah, Marion M. Stamp-Cole, Kimberly T. Perry, the Cefmetazole Study Group

**Affiliations:** 1 Department of Ob/Gyn/REI Temple University Hospital 3401 N. Broad Street Philadelphia PA 19140 USA; 2 University of Texas Medical Branch at Galveston TX USA; 3 University of California San Francisco General Hospital San Francisco CA USA; 4 University of New Mexico School of Medicine Albuquerque NM USA; 5 Upjohn Company Kalamazoo MI USA

## Abstract

*Objective:* The purpose of this study was to compare the clinical efficacy and safety of cefmetazole given by IV push with that of parenterally administered cefoxitin for the treatment of endometritis following cesarean delivery.

*Methods:* In a single-blind, multicenter, prospective, randomized study, 355 patients with endometritis after cesarean delivery were enrolled and received medication. Administered was either cefmetazole sodium, 2 g by IV push over 1 min q 8 h, or cefoxitin sodium, 2 g IV q 6 h in a 2:1 ratio. The patients were followed for clinical responses and side effects.

*Results:* The cure rate for cefmetazole was 89% and for cefoxitin it was 79% (*P* = 0.006). The adverse events were similar in both groups.

*Conclusions:* Cefmetazole was significantly more effective than cefoxitin in the treatment of endometritis following cesarean delivery.


Infectious Diseases in Obstetrics and Gynecology 3:28–33 (1995)
© 1995 Wiley-Liss, Inc.

### Single-Blind, Prospective, Randomized Study of Cefmetazole and Cefoxitin in the Treatment of Postcesarean Endometritis

**Ashwin Chatwani, Mark Martens, David A. Grimes, Molly Chatterjee, Melvin Noah, Marion M. Stamp-Cole, Kimberly T. Perry, and the Cefmetazole Study Group[1]**

*Temple University School of Medicine, Philadelphia, PA (A.C.), University of Texas Medical Branch at Galveston, TX (M.M.), University of California, San Francisco General Hospital, San Francisco, CA (D.A.G.), University of New Mexico School of Medicine, Albuquerque, NM (M.C.), and Upjohn Company, Kalamazoo, MI (M.N., M.M.S.-C., K.T.P.)*

### Abstract

***Objective:*** **The purpose of this study was to compare the clinical efficacy and safety of cefmetazole given by IV push with that of parenterally administered cefoxitin for the treatment of endometritis following cesarean delivery.**

***Methods:*** **In a single-blind, multicenter, prospective, randomized study, 355 patients with endometritis after cesarean delivery were enrolled and received medication. Administered was either cefmetazole sodium, 2 g by IV push over 1 min q 8 h, or cefoxitin sodium, 2 g IV q 6 h in a 2:1 ratio. The patients were followed for clinical responses and side effects.**

***Results:*** **The cure rate for cefmetazole was 89% and for cefoxitin it was 79% ($P = 0.006$). The adverse events were similar in both groups.**

***Conclusions:*** **Cefmetazole was significantly more effective than cefoxitin in the treatment of endometritis following cesarean delivery.** © 1995 Wiley-Liss, Inc.

Key words

Cesarean delivery, cephamycins, cephalosporins, bacteria

A major cause of maternal morbidity during the postpartum period is endometritis. Cesarean delivery is the most important clinical factor for the development of endometritis. The incidence of endometritis following cesarean delivery has ranged from 5 to 85%.[1] Endometritis is polymicrobial[2–4] in nature; therefore, antibiotic coverage often requires multiple agents to achieve adequate coverage. In an attempt to simplify the therapy, some investigators[5,6] have evaluated monotherapy with cephamycins.

Cephamycins are a group of parenterally administered cephalosporins characterized by an alpha-methoxy group at position 7. This configuration enables the cephamycins to be effective against a remarkable number of aerobic and anaerobic bacte-

**[1]Cefmetazole Study Group: Janet Arno, Indiana University Medical Center, Indianapolis, IN; James F. Beattie, Mount Carmel Medical Center, Columbus, OH; Bernard Gonik, University of Texas Health Sciences Center, Houston, TX; Randolph Hadley, University of South Alabama Medical Center, Mobile, AL; George Nelson and Charles Shipley, University of South Carolina School of Medicine, Columbia, SC; Francine Sinofsky, UMDNJ-Robert Wood Johnson Medical Center, New Brunswick, NJ; Edwin Thorpe, University of Tennessee Medical Center, Memphis, TN; Guillemo Valenzuela, San Bernardino County Hospital, San Bernardino, CA.**

**Address correspondence/reprint requests to Dr. Ashwin Chatwani, Department of Ob/Gyn/REI, Temple University Hospital, 3401 N. Broad Street, Philadelphia, PA 19140.**

Received September 28, 1994
Accepted February 14, 1995

ria. Studies comparing different cephamycins for prophylaxis and therapy have yielded similar efficacy and cure rates.[7–9]

With most regimens reporting similar cure rates for different cephamycins, the focus has shifted to the cost and ease of administration. A drug with a longer half-life requiring less frequent administration can result in considerable savings.[10,11] Cefmetazole, a new cephamycin, has a longer half-life than cefoxitin. It has also been demonstrated to be more active than cefoxitin against isolated species.[12–14]

With this in mind, we initiated this randomized, controlled trial to compare the clinical effectiveness and safety of cefmetazole vs. cefoxitin in the treatment of endometritis after cesarean delivery.

### MATERIALS AND METHODS

### Patients

The patients eligible for enrollment were hospitalized women, 16 years of age or older, with a presumptive diagnosis of endometritis after cesarean delivery. The diagnosis of endometritis was made by the clinical findings of temperature ≥38.3°C during the 1st 24 h after surgery or ≥38°C after the 1st 24-h period, fundal tenderness, adnexal tenderness, and purulent lochia. All findings were required for the enrollment of a patient in this study. Patients with symptoms suggesting other foci of infection, e.g., urinary tract or pulmonary system, were excluded from the study, as were patients who had received antibiotics, excluding antibiotic prophylaxis, within 48 h of recruitment in the study. Also excluded were patients with a history of hypersensitivity to penicillin or cephalosporins/cephamycins, neutropenia, liver or kidney dysfunction, or prior enrollment in this study.

### Study Design

The clinical protocol for this study was approved by the institutional review boards of the 12 participating centers. The centers were chosen from geographic areas so as to obtain a heterogeneous group of patients. A simple computer-generated randomization table was provided by the Upjohn Company for each site. A 2:1 allocation to cefmetazole vs. cefoxitin was used. Assuming that cefoxitin has a 95% cure rate, to detect a 5% difference with an 80% power, we needed approximately 350 cefmetazole subjects and 175 cefoxitin subjects. However, enrolling this number of patients was beyond the resources available for this investigation.

After each patient had given written informed consent, a physical examination was performed and the following laboratory studies were conducted: urinalysis, CBC with differential, prothrombin time, direct Coomb's test, alanine aminotransferase, aspartate aminotransferase, lactic dehydrogenase, alkaline phosphatase, total bilirubin, blood urea nitrogen, and serum creatinine. Cultures were obtained from the urine, uterus, and, if indicated, blood prior to treatment. The indications for blood culture were left to the discretion of each individual investigator. Uterine cultures were obtained by transcervical protected brush or endometrial aspiration curette. Cultures were obtained for facultative aerobes and anaerobes. For facultative aerobes, port-a-cul medium was inoculated onto sheep blood agar plates, MacConkey agar plates, chocolate agar plates, Martin-Lewis agar plates, and V-agar plates. For anaerobes, the medium was inoculated onto CDC anaerobic blood agar, CDC anaerobic laked blood agar with kanamycin and vancomycin, and prereduced PRAS brain heart infusion. The bacteria were isolated and identified, and their susceptibility to testing was determined by a serial agar dilution or by Kirby-Bauer disk diffusion.

Cefmetazole or cefoxitin was administered IV at a dosage of 2 g over 1 min q 8 h or 2 g by 30-min infusion q 6 h, respectively. A quick IV bolus route was chosen for cefmetazole to see if it was tolerated without an increase in local side effects. The study medication was continued for 24 h following the resolution of the signs and symptoms of endometritis. No discharge antibiotics were given to either group.

### Patient Evaluation

Over the course of treatment, which lasted 1–18 days, the patients were observed regarding their clinical status and potential adverse reactions. Specific questions were asked daily regarding any local discomfort at the IV site. Other factors included oral temperature evaluations every 4–6 h. The blood culture was repeated daily for a patient with a positive culture before treatment until it became negative. All clinical laboratory tests were repeated every 3 days while the patient was hospitalized and on the day of discharge. The uterine cultures were repeated only if a patient required a change in antibiotics.

A satisfactory clinical response to treatment was defined by the disappearance of any signs or symptoms of endometritis until the follow-up visit at 3–4 weeks. A clinical failure was defined as a temperature of ≥38°C on one or more occasions in a patient who had received the study medication for a minimum of 48 h, along with persistence or worsening of the signs and symptoms of endometritis that required changing the antimicrobial agents.

### Statistical Evaluation

For the demographic variables of a continuous nature, e.g., age, the treatment differences were tested using a 1-way analysis of variance (ANOVA) model, with treatment as the factor. Categorical demographic variables, e.g., race, and the proportion of patients reporting at least 1 medical event during the study were compared between groups using the Pearson chi-squared test for homogeneity of distribution. The number of days in the hospital until discharge was investigated using a 2-way ANOVA, with treatment, investigator, and investigator-by-treatment interaction as factors and investigators with ≤5 patients in either group pooled together. The Beslow-day test was used to check for investigator-by-treatment interaction for the primary efficacy variable. If the investigator-by-treatment interaction was not significant (using a more liberal value of $P > 0.10$), then the Cochran-Mantel-Haenszel test was used to determine the treatment effect. If the investigator-by-treatment interaction was significant ($P \leq 0.10$), then the investigators were not pooled and a chi-squared test was used to determine the treatment effect for each investigator ($P \leq 0.1$ was used for testing the investigator-by-treatment interaction to reduce the number of false positives, i.e., declaring that no interaction is present). The evaluability rate was compared between treatment groups using the Cochran-Mantel-Haenszel test, as described above. All statistical tests were 2-sided tests. Unless specifically noted, the results were reported as statistically significant if $P \leq 0.05$ was obtained.

### RESULTS

### Patients

Among the 12 participating institutions, a total of 382 patients were enrolled: 252 in the cefmetazole group and 130 in the cefoxitin group. Of the 382 randomized patients, the 377 who received medication (248 received cefmetazole and 129 received cefoxitin) were evaluable for safety. The initial protocol called for the enrollment of patients with any pelvic infection, but this was soon restricted to endometritis after cesarean delivery. Therefore, 22 patients without the aforementioned condition were excluded for the efficacy evaluation (16 in the cefmetazole group and 6 in the cefoxitin group). Thus, there were 355 patients available with endometritis for the efficacy evaluation (232 for cefmetazole and 123 for cefoxitin). The evaluability rate was not significantly different between treatment groups. The 2 groups were comparable with regard to age, weight, height, race, and initial temperature (Table 1).

**TABLE 1. Patient characteristics: Evaluable patients**

| Variable | Cefmetazole | Cefoxitin |
|---|---|---|
| Mean age ± SD (years) | 24.3 ± 5.6 | 24.3 ± 5.5 |
| Weight ± SD (Kg) | 78.3 ± 18.2 | 78.0 ± 18.4 |
| Height ± SD (cm) | 159.6 ± 12.5 | 160.9 ± 7.4 |
| Race (%) | | |
| White | 16.8 | 18.7 |
| Black | 41.0 | 46.3 |
| Oriental | 0.9 | 0 |
| Hispanic | 40.0 | 31.7 |
| Others | 1.3 | 3.3 |
| Initial temperature ± SD (°C) | 38.2 ± 0.7 | 38.2 ± 0.7 |

### Outcome

The number of days in the hospital ranged from 1 to 18. The overall means for the treatment groups were 5 days for the cefmetazole group and 5.4 days for the cefoxitin group ($P > 0.05$). There was a tendency for a longer hospital stay in the cefoxitin group. The number of days required in the hospital was comparable between treatment groups for investigators A.C., D.A.G., and M.M. However, the average hospital stay was significantly higher in the cefoxitin group (8.3 days) than in the cefmetazole group (5.5 days; $P = 0.006$) for the other investigators pooled together. Most patients (74%) defervesced within 1–3 days; the difference between treatment groups in days to defervescence was not significantly different.

TABLE 2. Clinical success rate: Evaluable patients

| | Cefmetazole | | Cefoxitin | | |
|---|---|---|---|---|---|
| | N | % | N | % | P |
| Success | 206 | 89 | 97 | 79 | 0.006 |
| Failure | 26 | 11 | 26 | 21 | |
| Total | 232 | 100 | 123 | 100 | |

The overall success rate for the cefmetazole group was 206/232 (89%) compared with 97/123 (79%) for the cefoxitin group ($P = 0.006$; Table 2).

Initial cultures were available from 237 patients (Table 3). An MIC of ≥64 μg/ml was considered resistant. In general, the MIC for cefmetazole was comparable with that of cefoxitin.

The overall frequency of adverse events was not significantly different between the 2 groups; 24% of patients in the cefmetazole group had 1 or more medical events compared with 23% of those treated with cefoxitin. Medical events relating to the gastrointestinal system were the most frequent, with 13/248 (5.3%) patients reported for cefmetazole and 10/129 (7.7%) for cefoxitin. Medication injection-site discomfort was reported in 12/248 (4.8%) patients in the cefmetazole group and 5/129 (3.9%) patients in the cefoxitin group. Allergic-type skin reactions were seen in 6/248 (2.4%) of those given cefmetazole, compared with 4/129 (3.1%) of those in the cefoxitin series. The frequency of adverse events was comparable among the investigators.

### Failures

Twenty-six failures were reported in the cefmetazole group and 26 in the cefoxitin group. The 23 patients in the cefmetazole group who continued to have fever spikes and evidence of uterine infection were deemed treatment failures. They subsequently responded to alternate antibiotic regimens, which included various combinations of clindamycin, gentamicin, ampicillin, cephalexin, doxycycline, and amoxicillin/calvulanate. One patient developed septic thrombophlebitis and recovered after heparin therapy. Two patients developed wound infections and required alternate antibiotics. In the cefoxitin group, 20 patients were deemed treatment failures and were subsequently treated with different antibiotics. One patient developed a wound infection and 2 patients were treated for presumed septic thrombophlebitis with heparin. One patient removed herself from the study because of a local reaction. In addition, 2 patients had satisfactory responses to the initial protocol treatment but had relapses requiring additional antibiotics 1 week after discharge.

### DISCUSSION

The introduction of cephamycin antibiotics has allowed monotherapy of various obstetric and gynecologic infections. Monotherapy has many advantages. First, and most important to the patient and physician, is the fact that combination therapy involves the possible toxic effects of 2 or more drugs. Furthermore, there is an ease of administration with monotherapy. It can be less expensive because of the savings of not mixing antibiotics and using fewer IV tubins. In the era of health care moving to managed care, less expensive therapy does not go unnoticed by health-care insurance administrators.

Cefoxitin was the 1st cephamycin agent introduced and has been the most widely used. Clinical cure rates with cefoxitin have ranged from 84 to 97%.[5,15] However, the major disadvantage of cefoxitin is its pharmacokinetics. The half-life of cefoxitin has been reported to be 30–60 min,[16,17] requiring administration q 6 h. Less frequent administration is desirable because it is cheaper and less time-consuming for pharmacy and nursing personnel.

Cefmetazole is a new cephamycin with the advantage of a longer half-life, 90 min,[17,18] allowing administration q 8–12 h.[19] The replacement of a cephamycin with less frequent dosing can result in a 35% savings in the annual cost of antibiotics.[10,11] In addition, cefmetazole is significantly more stable than cefoxitin with essentially no hydrolysis over the 24-h incubation period.[20] It has been shown that, because of this longer half-life and more stability, the tissue level of cefmetazole is significantly higher than that of cefoxitin.[21] Concentrations of cefmetazole persist longer in serum and tissue than those of cefoxitin do, suggesting an advantage for the treatment of endometritis. Even though cefmetazole and cefoxitin have similar in vitro microbiology, the pharmacokinetic differences give superiority to cefmetazole.

We have shown that endometritis after cesarean delivery can be effectively treated with cefmetazole therapy, achieving a clinical success rate of 89%,

**TABLE 3. Cefmetazole and Cefoxitin MIC**

| Organisms | No. of isolates | Cefmetazole MIC range (μg/ml) | Cefmetazole % Susceptible | Cefoxitin MIC range (μg/ml) | Cefoxitin % Susceptible |
|---|---|---|---|---|---|
| Gram-positive aerobes | | | | | |
| *Enterococcus* species | 93 | 32–512 | <1 | 32–512 | <1 |
| *Streptococcus* | | | | | |
| Group B streptococci | 62 | 0.25–32 | 100 | 0.5–4.0 | 100 |
| Other species | 28 | 0.125–64 | 96 | 0.5–64 | 93 |
| Alpha hemolytic streptococci | 19 | 0.5–32 | 100 | 2–16 | 100 |
| *Staphylococcus* | | | | | |
| *Staph. epidermidis* | 42 | 0.5–32 | 100 | 0.125–32 | 100 |
| *Staph.*-coagulase negative | 21 | 1–16 | 100 | 1–32 | 100 |
| *Corynebacterium* species | 11 | 0.125–8 | 100 | 0.125–16 | 100 |
| Gram-negative aerobes | | | | | |
| *Escherichia coli* | 38 | 0.25–4 | 100 | 1–16 | 100 |
| *Enterobacter* species | 18 | 8–512 | 12.5 | 16–512 | 12.5 |
| *Klebsiella pneumoniae* | 15 | 0.25–4 | 100 | 0.5–8 | 100 |
| *Proteus* species | 13 | 0.5–4 | 100 | 1–4 | 100 |
| *Pseudomonas* | 10 | 64–512 | 0 | 64–512 | 0 |
| *Neisseria gonorrhoeae* | 9 | 0.5–8 | 100 | 0.5–2 | 100 |
| *Acinetobacter* species | 9 | 32–512 | <1 | 32–512 | <1 |
| Anaerobes | | | | | |
| *Bacteroides* | | | | | |
| *B. bivius* | 47 | 0.25–64 | 94 | 0.25–64 | 94 |
| *B. fragilis* | 18 | 0.5–16 | 100 | 1–16 | 100 |
| *B. melaninogenicus* | 16 | 0.12–8 | 100 | 0.25–8 | 100 |
| Other species | | | | | |
| *Peptostreptococcus* species | 45 | 0.5–32 | 95 | 0.125–32 | 95 |
| *Clostridium* species | 5 | 0.25–16 | 100 | 1–64 | 80 |
| *Fusobacterium* | 4 | 0.5–8 | 100 | 0.5–8 | 100 |
| *Gardnerella vaginalis* | 13 | 0.125–0.5 | 100 | 0.5–2 | 100 |

and 79% for cefoxitin ($P = 0.006$). Cefmetazole is more effective than cefoxitin because of the previously mentioned differences in pharmacokinetics. Cefmetazole was given by IV bolus infusion rather than by constant infusion over a period of time. The highest serum and tissue levels of antibiotics are reached by the bolus injection.[16,22] The administration of cefmetazole by 1-min IV push was not associated with any increase in local reactions. This finding is particularly attractive as it eliminates the cost of infusion bags.

Even though the clinical cure rates were significantly different between the groups, the time to defervescence was not statistically different in the 2 groups. The reasons for this outcome are not clear.

In summary, in the evaluation of an antibiotic in this cost-containment era, the cost effectiveness of treatment, including the economic impact of making such changes, must be considered. This evaluation can be simplified by assigning weights to major criteria, i.e., spectrum of activity, pharmacokinetics, frequency and ease of administration, adverse reactions, costs, and stability. Under this analysis, cefmetazole received a much higher score than cefoxitin did.[23]

### REFERENCES

1. Duff P: Pathophysiology and management of post-cesarean endometritis. Obstet Gynecol 67:269–276, 1986.
2. Sweet RL: Anaerobic infections of the female genital tract. Am J Obstet Gynecol 122:891–895, 1975.
3. Gorbach S, Bartlett JG: Anaerobic infections: Old myths and new realities. J Infect Dis 130:307–312, 1974.
4. Escenbach DA, Buchanan TM, Pollock HM, et al.: Polymicrobial etiology of acute pelvic inflammatory disease. N Engl J Med 293:166–170, 1975.
5. Sweet RL, Ledger WJ: Cefoxitin: Single-agent treatment of mixed aerobic-anaerobic pelvic infections. Obstet Gynecol 54:193–198, 1979.
6. Faro S, Sanders CV, Aldridge KE: Use of single-agent antimicrobial therapy in the treatment of polymicrobial female pelvic infections. Obstet Gynecol 60:232–236, 1982.

7. Shimada J, Hayashi Y, Nakamura K: Cefmetazole: Clinical evaluation of efficacy and safety in Japan. Drugs Exp Clin Res 11:181–187, 1985.
8. MacGregor RR, Graziani AL, Samuels P: Randomized double blind study of cefotetan and cefoxitin in post-cesarean section endometritis. Am J Obstet Gynecol 167: 139–143, 1992.
9. Berne TV, Yellin EA, Appleman MD, et al.: Controlled comparison of cefmetazole with cefoxitin for prophylaxis in elective cholecystectomy. Surg Gynecol Obstet 170: 137–140, 1990.
10. Martinusen S, Chew D, Frighetto L, Bunz D, Stiver H, Jewesson PJ: Comparison of cefoxitin and ceftizoxime in a hospital therapeutic interchange program. Can Med Assoc J 148(7):1161–1169, 1993.
11. Wright DB: Antimicrobial formulary management: A case study in a teaching hospital. Pharmacotherapy 11: 27s–31s, 1991.
12. Jones RN: Review of the in-vitro spectrum of characteristics of cefmetazole. J Antimicrob Chemother 23(Suppl D):1–6, 1989.
13. Frank E, Phillips SL, Gupta T, et al.: Treatment of skin and soft tissue infections: A comparative study of cefmetazole and cefoxitin. J Antimicrob Chemother 23(Suppl D):55–60, 1989.
14. Roy S, Wilkins J, Galafi E, et al.: Comparative efficacy and safety of cefmetazole or cefoxitin in the prevention of postoperative infection following vaginal and abdominal hysterectomy. J Antimicrob Chemother 23(Suppl D): 109–117, 1989.
15. Hager WD, MacDaniel PS: Treatment of serious obstetric and gynecologic infections with cefoxitin. J Reprod Med 28:337–340, 1983.
16. Ledger W: Antimicrobial agents. In: Infection in Female. 2nd ed. Philadelphia: Lea & Febiger, p 95, 1986.
17. Jones RN, Barry AL, Fuchs PC, et al.: Antimicrobial activity of cefmetazole and recommendations for susceptibility testing by disk diffusion, dilution and anaerobic methods. J Clin Microbiol 24:1055–1061, 1986.
18. Berriere SL: Formulary consideration for the 7-methoxy cephalosporins (cephamycins). Hosp Ther 15:54–60, 1990.
19. Brook I: Use of cephalosporins for prophylaxis and therapy of polymicrobial infection in mice. Antimicrob Agents Chemother 37(7):1531–1535, 1993.
20. Kasai T, Hara T, Tamura A: In vitro and in vivo activities of a novel cephamycin MT-141 against the *Bacteroides fragilis* group in comparison with six cephem antibiotics. J Antimicrob Chemother 15:707–709, 1985.
21. DiPiro JT, Connors JE, Bowen TA Jr, et al.: Comparative intraoperative concentration of two cephalosporins with activity against anaerobic bacteria. J Antimicrob Chemother 23:89–91, 1989.
22. Barza M, Brusch J, Bergerson MG, Weinstein L: Penetration of antibiotics into fibrin loci in vitro. III. Intermittent vs. continuous infusion and the effect of probenecid. J Infect Dis 129:73–76, 1974.
23. Jones RN: Role of new cephamycins in the management of obstetrics and gynecologic infections. J Reprod Med 35(11):1070–1077, 1990.
